# LivePhantom: Retrieving Virtual World Light Data to Real Environments

**DOI:** 10.1371/journal.pone.0166424

**Published:** 2016-12-08

**Authors:** Hoshang Kolivand, Mark Billinghurst, Mohd Shahrizal Sunar

**Affiliations:** 1 MaGIC-X (Media and Games Innovation Centre of Excellence) UTM-IRDA Digital Media Centre Universiti Teknologi Malaysia, 81310 Skudai, Johor, Malaysia; 2 School of Information Technology and Mathematical Sciences, University of South Australia, Adelaide SA 5001, Australia; West Virginia University, UNITED STATES

## Abstract

To achieve realistic Augmented Reality (AR), shadows play an important role in creating a 3D impression of a scene. Casting virtual shadows on real and virtual objects is one of the topics of research being conducted in this area. In this paper, we propose a new method for creating complex AR indoor scenes using real time depth detection to exert virtual shadows on virtual and real environments. A Kinect camera was used to produce a depth map for the physical scene mixing into a single real-time transparent tacit surface. Once this is created, the camera’s position can be tracked from the reconstructed 3D scene. Real objects are represented by virtual object phantoms in the AR scene enabling users holding a webcam and a standard Kinect camera to capture and reconstruct environments simultaneously. The tracking capability of the algorithm is shown and the findings are assessed drawing upon qualitative and quantitative methods making comparisons with previous AR phantom generation applications. The results demonstrate the robustness of the technique for realistic indoor rendering in AR systems.

## Introduction

Augmented Reality (AR) involves the integration of virtual content into real environments [1]. In AR applications, the virtual objects appear more realistic through the use of shadows and lighting [2] [3] enabling the virtual content to be seamlessly blended with the real world [4, 5]. One way to do this is to have virtual objects lit from lighting in the real world, and to get virtual objects to cast shadows onto real objects.

Shadows play a major role in the production of realistic AR systems. This is because shadows produce the perceptual illusion of the virtual in real world empowering the observer to determine the distance between various objects enhancing object complexity and displaying more realistic environments. Shadows can also be employed to yield the light position among the lighting information rendering it more believable than what is the case with the virtual object in the real world.

Many algorithms have been developed for shadow generation. However, these algorithms may not be suitable for the real time requirements of AR applications. For example, shadow volumes [6] are sufficiently accurate but they are geometrically-based and require extensive calculations. In this paper, we present a new algorithm to achieve real-time realistic virtual shadows in real environments. This algorithm is based on reconstructing the physical scene as phantoms in the Augmented Realty system and capturing the dense data of 3D models of physical scenes using our technique (which we called LivePhantom) such that a mesh, as phantoms, is created in AR to receive shadows of augmented objects in real environments. The remainder of this paper is as follows: Section 2 provides an overview of previous research on reconstruction and shadow generation in AR is presented. Section 3 presents the AR setup supported by a Kinect camera describing LivePhantom’s capabilities in detail. Section 4 covers the results and discussion including the topic of casting virtual shadows on real environments. This section also takes up the question of evaluating different components of the pipeline as results concerning the four different scenes. The paper ends with a brief conclusion and suggestions for future developments to overcome the remaining issues.

## Related Works

Shadows are from among the most salient factors contributing to the AR system realism whose subject has been researched for the last fifteen years [1]. Shadows help realise the relative distance of objects in a scene not only for stratified real world, e.g., underground [7] but also for virtual environments such as virtual molecula [8], virtual surgery [9], virtual hydrological environments [10] and virtual city [11, 12]. Shadows also help reveal the complexity of objects. Besides, shadows improve the user experience [13] of human-computer-interaction, e.g., on smartphone [14, 15] and the AR games based on it [16]. Without shadows, the distance and complexity of objects are almost vague especially in Augmented Reality where realistic virtual objects indistinguishable from the real ones are required.

Much research has been conducted on how AR environment shadows can be used and enhanced [17] [18] [3]. Research has also focused on the virtual shadow enhancement rendering objects increasingly realistic in outdoor AR [19] [20] considering real and virtual lighting interaction. The daytime illumination interaction of sky colour onto virtual environments can be regarded as the most recent research further realizing the AR system in spite of real environment shadows [21]

### 0.1 Shadows and Pre-Created Phantoms in AR

Recently, attempts have been made to employ Augmented Reality systems to produce shadows for virtual objects on flat surfaces so as not to reveal the absence of flexible soft and hard shadows on real and virtual objects [22] [23] [24] [25] [26].

Shadow volumes have been utilized in AR to produce shadows on real objects using an algorithm where a phantom model is produced to act as a real object [18]. In this regard, the outline of both virtual objects and phantom needs to be recognized. Phantoms were capable of receiving the virtual shadows. Advanced generation of 3D software phantoms is among the major issues in this technique. The method is not cost-effective; firstly due to the 3D phantoms in 3D software and secondly for the implementation of shadow volumes.

Shadow mapping was initially used in AR systems by Sugano et al. [27] through phantom object pre-creation so as to cast virtual shadows on real environments. The researchers investigated the advantages associated with AR system shadows in place of AR shadow generation.

A soft shadow technique was introduced by Supan et al. [28]. A shadow dome was employed casting virtual light sources to produce the output from environment shadowing. Among the advantages associated from this technique one can refer to seamless virtual scenario integration, shadowing based on images, provision of three setups, and the absence of pre-processed data. Nevertheless, research is not supportive of casting shadows on the virtual objects.

Dynamic range environment maps were utilized by Madsen and Laursen [29] to show real illumination albeit in stereo disparity images where they concentrated on shadow detection via a camera capable of recognizing location.

A soft shadow using projection shadows on flat surfaces was employed by Jensen et al. [30] under real light conditions while discounting other objects’ shadows. Nowrouzezahrai et al. [31] investigated light factorization in the case of AR augmented mixed-frequency shadows so as to reinforce the realism concentrating on indoor rendering shadow generation despite flat surface shadow casting.

Convolution Shadow Maps (CoSMs) [32] are one of the improved shadow algorithms utilized by Aittala [33] to generate AR soft shadows drawing upon both fast summed area tables and mip-map filtering [34] to further reinforce blurring via variable radii. Under these conditions, virtual shadow casting on real objects was not considered.

Madsen et al. [35] introduced a method whereby virtual shadows are generated on real objects via colour imagery predicting AR outdoor illumination conditions with reference to dynamic shadow detection. The researchers employed shadow volumes to produce virtual shadows. Direct illumination from the sun and the sky from dynamic shadow pixel values under live video conditions were considered in this case. Castro et al. [19] employed filtering methods including Percentage Closer Filtering (PCF) [36] and Variance Shadow Maps (VSM) [37] to produce shadows without interactions between virtual and real objects.

AR-related outdoor illumination conditions were predicted by Madsen and Lal [3] in terms of dynamic shadow detection drawing upon shadow volumes to produce virtual shadows. Direct illumination from the sun and the sky from dynamic shadow pixel values in live videos were considered in this case.

A soft shadow technique with less aliasing was introduced by Castro et al. [19]. The researchers assigned a fixed distance to the marker albeit using only one camera. The method undertakes sphere mapping [38]choosing one or a few light sources best representing the scene. This is especially salient due to hardware limitations associated with mobile devices. Nevertheless, the procedure has disadvantages related to self-shadowing and soft shadowing. The researchers employed filtering methods including PCF [36] and VSMs [37] to produce semi-soft shadows.

Integration of shadows and sky colour with respect to the sun’s position in AR is employed by Kolivand and Sunar [21, 39, 40] for outdoor rendering. The effect of the sky colour on the augmented objects during a day takes the location, date, and time into account to enhance the realism of outdoor rendering in AR systems.

Recent research on shadows in augmented realty includes the one conducted by Nowrouzezahrai et al. [31] who applied light factorization for augmented frequency shadows in AR environments to enhance the realism. Lighting is the main factor which enhances the realism. Compared to the present work there is no focusing of shadows onto real objects. To the best of our knowledge [18] [27], [31], [19], [3] and somehow [35] are the prominent works on shadows in augmented reality but they pay no attention to virtual shadows in real environments except [18] and [27] who employed pre-reconstruction of real objects.

As it is shown, most current systems for showing virtual shadows on real objects are based on static objects and fixed environments. These systems have their major limitations meaning that the objects in the real world could not be moved. In contrast, in our system real objects can be moved and the virtual shadows would adjust to their movement accordingly which has been achieved through the use of real time environment modeling.

### 0.2 Real-Time Environment Modeling

Numerous computer graphic techniques [41] [42] have been designed for the purpose of reconstructing physical scenes [43] [44]. The present research considers real time modeling via depth cameras such as the Microsoft Kinect. KinectFusion is presented by Izadi et al. [44] for reconstructing a mesh of objects in real-time using the Depth sensors to track 3D pose of the Kinect camera to generate 3D models for real scenes. The method relies on GPU to segment objects and to interact with the user.

Newcombe et al. [45] investigated indoor environment real-time mapping with the aid of a Kinect camera to reconstruct the geometry of the scene. Changing the position of the camera, the researchers fused both depth data from the sensors to reconstruct the captured environments.

Lack of moving volume in space is a shortcoming for KinectFusion [44] which Roth and Vona [46] tried to address by proposing a moving volume KinectFusion algorithm. The algorithm translates and rotates the volume when the camera moves. Thus, it is feasible for mobile robotics to provide visual odometery of real scenes.

Keller et al. [47] extended KinectFusion [44] and worked on online 3D reconstruction in dynamic scenes using point-based fusion. They used a moving sensor to collect depth measurements for a single model which refines it continuously.

In sum, all the reconstruction techniques mentioned here are based on the [48] [49] and [50] which we also have taken into account the reconstruction of the Phantoms.

### 0.3 Realism Issues in AR

There are many different issues influencing the perceived realism of 3D generated objects in computer graphics. Realism refers to some sort of measurement for the subjective difference between a real 3D environment and a 3D scene generated from computers [51]. Quantifying such measurement is not easy, since it is difficult to determine if a computer generated scene is the same as a real one.

One of the other issues with AR, is the need for exact illumination with respect to the environments to make the system maximally realistic [52] [35] [53] [54] [55].

Some researchers have explored the problem of virtual shadows in AR scenes. However, the majority of this work does not support casting and receiving the virtual shadows on real environments in real-time. The present research contributes to the literature on the topic in the following ways:

(1) Real-time reconstruction of real scenes, (2) Generation of real-time phantoms for any AR systems (3) Presenting the literature with a method for casting virtual shadows on the real environments, and (4)Application of PCF [36], Cascade Shadow Maps (CSMs) [56] and (Hybrid Shadow Maps) HSMs [57] in AR. Although, casting virtual shadows on real environments has been studied for more than a decade, it suffers from real-time reconstruction of real environments [18] [58] [33] [19] [3]thus requiring a significant improvement to be implemented in real-time.

## LivePhantom

This section takes up the question of various AR pipeline components and how these can generate virtual shadows being cast on real environments to create realistic AR systems. The virtual environments were initially modeled using 3D software such as 3D Studio Max. Subsequently, they were augmented onto the scene through a marker based tracking technique. The Augmented Reality system utilized the Metaio SDK [59] and the Unity3D game engine for rendering [60] for tracking purposes. In the virtual scene, a virtual light had to be located in the environment to control the shadow positioning. In the present research, shadows are produced using common techniques so as to demonstrate the capability of the system to simultaneously produce virtual shadows on virtual and real objects. The LivePhantom is utilized to reconstruct the physical scene in the form of a 3D model located in live video using a simple webcam embedded with a Kinect camera. Phantom rendering is performed using transparency so that the real objects being covered are seen. The phantoms were employed to represent virtual object shadows on real object faces.

**Fig 1:**
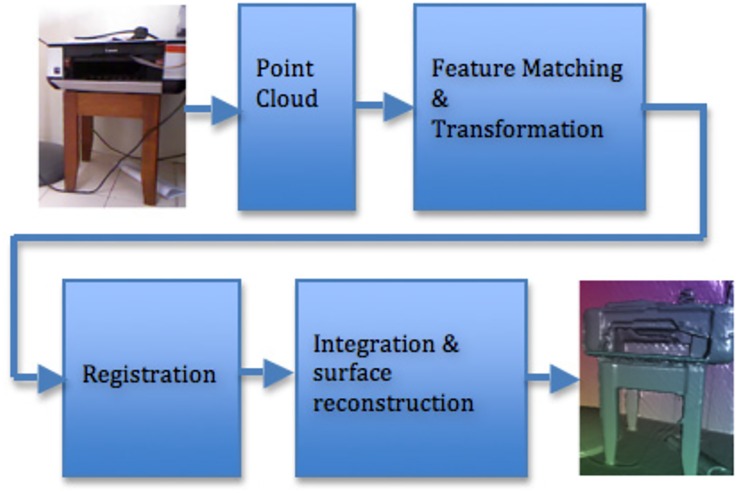
Pipeline of reconstructing 3D objects from a real scene.

The LivePhantom technique is used for capturing the real environment, AR tracking, reconstructing the real scene as phantoms, and generating shadows in AR. The pipeline is illustrated in Fig 1.

### 0.4 Capturing

Capturing the real environments is the first stage of the pipeline. To produce virtual shadows on real environments, the phantom which is similar to the real one needs to be generated. To do this, the Kinect camera is used to reconstruct real scenes in real-time.

The Kinect camera can be utilized to capture the real environment using six degrees of freedom leading to a simple real-time depth map having a point cloud. The noisy data must be refined to simple and sufficiently accurate coherent data which is undertaken through removal of neighbouring surfaces having less angles between their normalized normal vectors than a deem degree. If $N_{s_{p}}$ is the normalized vector of surface *s*_*p*_ and $N_{s_{q}}$ is the normalized vector of surface *s*_*q*_ which is the neighbouring vector of *s*_*p*_ and $|N_{s_{p}}-N_{s_{q}}|\;<d$ then *s*_*q*_ will be replaced by *s*_*p*_. Parameter *d* is flexible in that it reveals different accuracies for the phantoms between 0% and 15% at the first stage. To produce a complete point cloud, the real scene needs to be captured from different viewpoints and the data needs to be fused together.

There are two ways to reconstruct a more accurate and complete object; (1) capturing the object from a number of different viewpoints, and (2) fixing the Kinect camera and rotating the object in front of it. In the second method, the object fills the majority of depth map and so is better when a single object needs to be reconstructed.

Camera tracking is the next step after capturing the environments and generating the raw depth map is the next step. This is described in more detail in the following section.

### 0.5 Tracking

Camera tracking is based on the Interactive Closest Point (ICP) algorithm of Besl and McKay [48], as described below:

**Algorithm 1**. 3D reconstruction tracking

**Step 1**. *Create the point cloud*

**Step 2**. *Set the line segments between the points*

**Step 3**. *Produce the implicit curve equations like f(x, y, z)* = 0

**Step 4**. *Parametric the curves like (x(p), y(p), z(p))*

**Step 5**. *Calculate the triangles to be the surface*

**Step 6**. *Produce the implicit surface equations like g_f_(x, y, z)*

**Step 7**. *Parametric the surfaces (x(p, q), y(p, q), z(p, q))*

If *C* is the set of points with *N*_*c*_ points, the distance of point *p* to the set of *C* is:

$$\begin{array}{c} d\left(p,C\right)=min\;d\left(p,c_{i}\right),\;\;1\leq i\leq N_{c} \end{array}$$ (1)

Let *s* for segmenting two points *p* and *q*, then $S=\;{⋃}_{i=1}^{N_{l}}\left\{s_{i}\right\}$ all segmentations of *C*. If *ω* is a triangle between three points *p*_1_, *p*_2_, *p*_3_ and $\Omega =\;{⋃}_{i=1}^{N_{s}}\left\{\omega_{i}\right\}$ then the distance between *p* and *ω* could be calculated by [48]:

$$\begin{array}{c} d\left(p,\Omega \right)=min\;d\left(p,\omega_{i}\right),\;\;i\in \left\{1,..,N_{s}\right\} \end{array}$$ (2)

where

$$\begin{array}{c} d(p,q)=||\lambda p+(1-\lambda )q||,\;0\leq \lambda \leq 1 \end{array}$$ (3)

And finally the distance metric of *p* and shape *χ* will be calculated by:

$$\begin{array}{c} d\left(p,\chi \right)=min\;\overset{N_{\chi }}{\underset{i=1}{⋃}}\left|\right|x_{i}-p\left|\right| \end{array}$$ (4)

Now by calculating this method, all of the closest points will be determined which is denoted by *Y*:

$$\begin{array}{c} Y=\overset{N_{\chi }}{\underset{i=1}{⋃}}\overset{N_{c}}{\underset{j=1}{⋃}}\left|\right|x_{i}-p_{j}\left|\right| \end{array}$$ (5)

The complete registration vector is *q* = [*q*_*R*_|*q*_*T*_]^*t*^ where *R* is a rotation matrix and *T* is translation matrix. Thus:

$$\begin{array}{c} q_{T}=\mu_{x}-ℜ\left(q_{R}\right)\mu_{p} \end{array}$$ (6)

where *μ*_*x*_ is the centre of mass of measured point set *χ* and same for point set *P* with *μ*_*p*_. ℜ is the 3 × 3 rotation matrix created by a unit rotation quaternion [50].

The error between the two corresponding points in a rotation of *R* in translation *T* could be estimated by

$$\begin{array}{c} Error=\sum_{i=1}^{N_{c}}\left|\right|c_{i}R+T-\chi_{i}\left|\right| \end{array}$$ (7)

Finally, iterating the registration for *P*_*k*+1_ = *q*_*k*_(*P*_0_) is applied until *d*_*k*_ − *d*_*k*+1_ < *ε*.

Fusing the point clouds to create the required mesh involves the ICP algorithm [48]. This fuses the new depth frame with the current one by approximating a single transform that is closely matched with the current depth frame.

### 0.6 Surface Reconstructing

The proposed method makes it possible to employ a standard Kinect camera having an ordinary webcam to reconstruct the captured 3D objects as an AR system phantom. Any uncomplicated and minimal displacement of Kinect produces a separate viewpoint rendering the reconstruction objects more precise. Integrating different captured views from numerous viewpoints leads to increasingly precise phantoms but more extensive. To prevent this situation, the Kinect camera should be fixed. Otherwise, the real objects need to be moved. Another webcam provides the users the chance to observe the augmented objects from numerous viewpoints provided the objects had already been captured using the Kinect camera.

The most challenging part of casting shadows on real environments is in the case of real-time and complex environments. Creating phantoms using 3D software does not support a complex environment and they cannot be implemented in real-time rendering. That is, it is not a real-time reconstruction if it is done before-hand.

**Fig 2:**
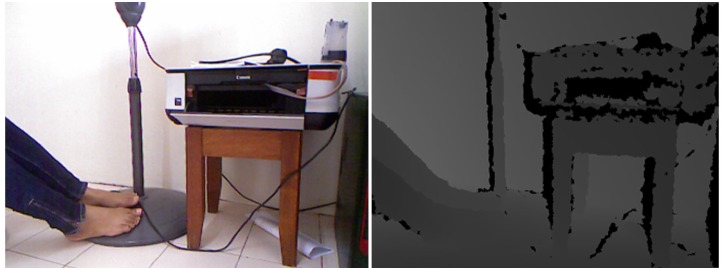
left: A real environment (RGB mode), right: row depth data.

To construct the 3D object from the depth map as can be seen in Fig 2, deem that for a pixel *p* = (*x*, *y*) at the time of *t* the depth is *D*_*t*_(*p*). The specific 3D vertex in the Kinect camera coordinates will be:

$$\begin{array}{c} v_{t}\left(p\right)=\frac{D_{t}\left(p\right)}{K[p,1]} \end{array}$$ (8)

where *K* is the calibration matrix of the Kinect infrared camera. For each vertex the normal vector is calculated using:

$$\begin{array}{c} n_{t}\left(p\right)=\left(v_{t}\left(x+1,y\right)-v_{t}\left(x,y\right)\right)\times \left(v_{t}\left(x,y+1\right)-v_{t}\left(x,y\right)\right) \end{array}$$ (9)

The main factors for evaluating the quality of the point cloud, are point density and accuracy [61]. The sensor, measurement setup, and properties of the object surface are the main sources of imperfection and error. Sensor error is mostly caused by inadequate calibration while imaging geometry and lighting condition are due to measurement setup error. Properties of the objects can also impact the measurement of the points.

The resolution of the Kinect and the pixel size of the disparity image are important for calculating the point spacing of the depth. Therefore, there is an inverse relationship between the point density and sensor distance.

#### 0.6.1 Mesh Generation

**Fig 3:**
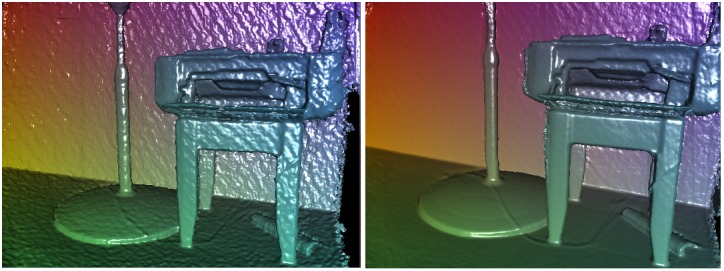
Left: 3D constructed surface from a single viewpoint, Right: 3D constructed surface form a multi-viewpoint.

Generating a 3D mesh from the set of point cloud is employed by connectivity between neighbouring points, as discussed earlier. Of course, capturing the physical scene from different points of view can create sufficiently high quality, but there is no need to be more accurate due to invisibility of the phantoms. The difference between capturing from a single viewpoint and capturing from more viewpoints is illustrated in Fig 3. Fig 3 (left) is the generated surface with capturing from a single point of view that is not accurate enough but can be used for the purpose of this study. Fig 3 (right) is the 3D surface which is captured from different viewpoints.

**Fig 4:**
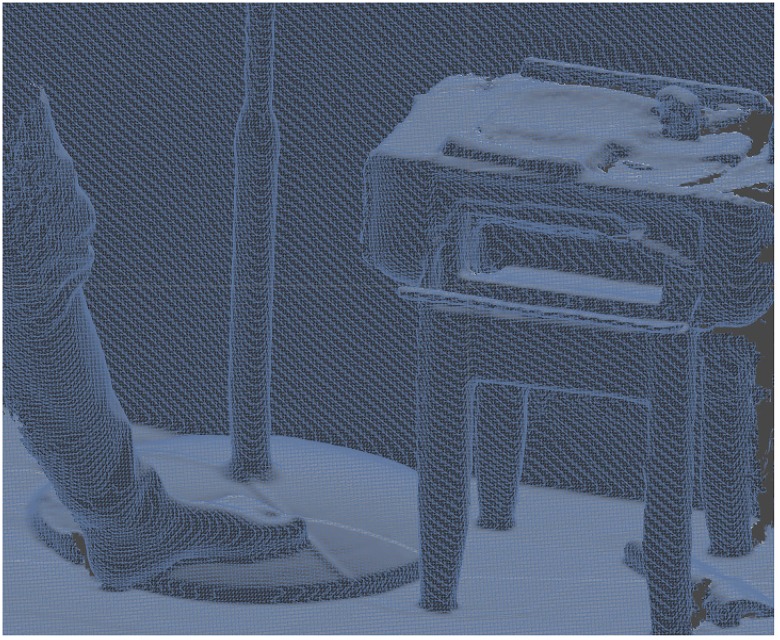
Generated mesh which could be used in any other 3D software.

**Fig 5:**
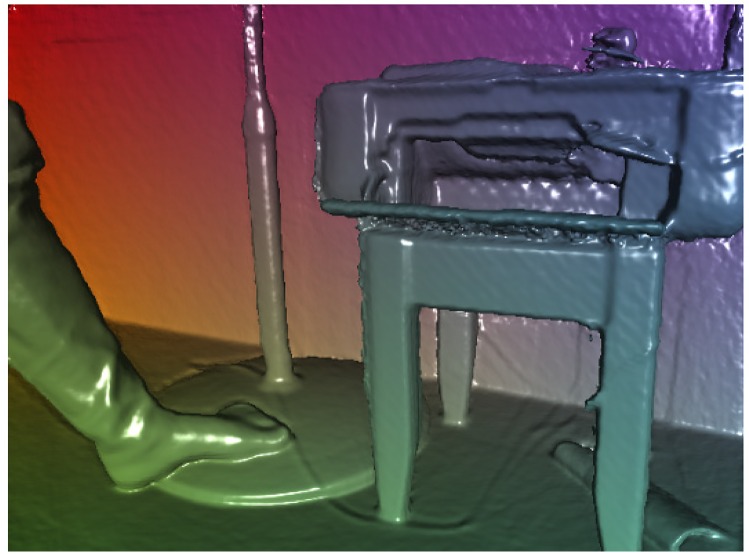
3D model generated from LivePhantom showing surface normals.

The 3D mesh reconstructs within milliseconds (less than 28 ms). This mesh is needed not only to receive the shadows but also to apply collider for having the interaction between virtual objects and real environments. Fig 4 shows the mesh that is generated based on the depth map. Fig 5 is a surface which is generated based on the mesh in Fig 4.

#### 0.6.2 Phantom Orientation

The orientation of the reconstructed 3D environment is not aligned and does not fit the original captured data from the AR webcam when it moves away from the Kinect camera. The reconstructed data need to be adjusted during the rendering. In an indoor case such as a normal room or office the orientation is adjusted with (*α*, *β*, *γ*) in the direction of (*x*, *y*, *z*) where *α*, *β* and *γ* are the coordinates of the marker. Orientation needs to be adjusted in real-time.

### 0.7 Shadow Generation

In this section, we describe our approach for generating semi-soft shadows for AR systems. To show the step-by-step process to achieve realistic AR systems, shadows are employed after generating the conventional AR systems. HSMs [57] are suitable to create semi-soft shadows on other virtual objects due to image basing and low calculation load.

Hybrid shadow maps are constructed based on shadow mapping algorithm. The view frustum is split into *m* partitions for controlling the resolution of each part. Multiple layers are taken into account to store each partition in an independent layer. Resolution of each layer is set in order to increase the quality of shadow and present the high-cost rendering. Logarithm function is selected to distribute layer situation to enhance shadow quality. The algorithm is summarised as follows:

**Algorithm 2** Hybrid Shadow Maps

**Step 1**. *Render the entire scene from point of view and store the mean and mean squared of depth distribution*.

**Step 2**. *Render the entire scene again from the light source’s point and store the mean and mean squared of depth distribution*.

**Step 3**. *Split frustum point of view into multiple partitions, depending on the size of the scene using logarithm function starting from the nearest object according to the camera’s point of view*.

View frustum splitting starts from the first object in the virtual scene. This idea allows the GPU to act independently of those parts of the scene that are outside any rendering contribution. This technique, in addition to accelerating the algorithm, substantially reduces the number of layers.

The partitions developed through splitting the view frustum using logarithm function are not uniform; some parts of the scene closer to the first object are divided into small partitions, whereas others do not require much resolution as they are located in large partitions. Moreover, the logarithm function contributes to high speed rendering. It should be noted that most of the objects are located around the centre of the cone in the view frustums.

The initial implementation has started from Metaio with multiple markers loop functioning as a starting point, then a function to render a Metaio GL scene is used passing the geometry of the scene as function parameters. The GL scene function calls another GL display method in the Metaio GL. The method calls the initializations of the scene and the display loop determining the geometry of the virtual scene. Knowing that the shadows, depending on HSMs, of each object are rendered within the scene itself, it would make it easier for a programmer to render the shadows in AR environments. Moreover, to show the realistic interaction between real and virtual objects, simulated primitive alpha objects resembling real objects are tracked in the same position, location and orientation of the real ones as mentioned in tracking part.

## Results and Discussion

As for real-time rendering, the LivePhantom is sufficiently accurate, Fig 5. The wire and the fingers for each printer section can clearly be observed. Nevertheless, the wire on the leg of the fan is less than 5 mm thick being observed accurately.

Generating shadows on other objects is another subject considered in this research. No extra stages are required to generate virtual shadows on virtual objects through implementing conventional Shadow Maps [62], Percentage Closer Filtering (PCF) [36], CSMs [56] and HSMs [57]. HSMs are based on shadow maps. Therefore, casting the virtual shadows on other objects is the main ability of this category of shadow generation techniques.

### 0.8 Shadow Evaluation

**Fig 6:**
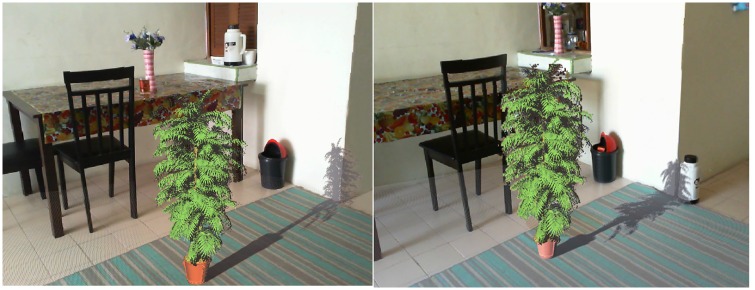
HSMs on real environments, left: virtual shadow on a corner of room, right: virtual shadows on two real objects(wall and vacuum flask).

Fig 6 (left) shows an AR system where the shadow of the virtual objects is cast on a real wall. Fig 6 (right) shows the virtual shadows on two real objects. In these pictures the wall and the vacuum flask are real while the plant is virtual. The light position can be readily adjusted using keyboard as is the case with location of virtual objects.

To comparing the current work with other shadow techniques in augmented reality, four latest techniques, Shadow Maps, PCF, CSMs and HSMs are chosen due to generating realistic shadows.

**Fig 7:**
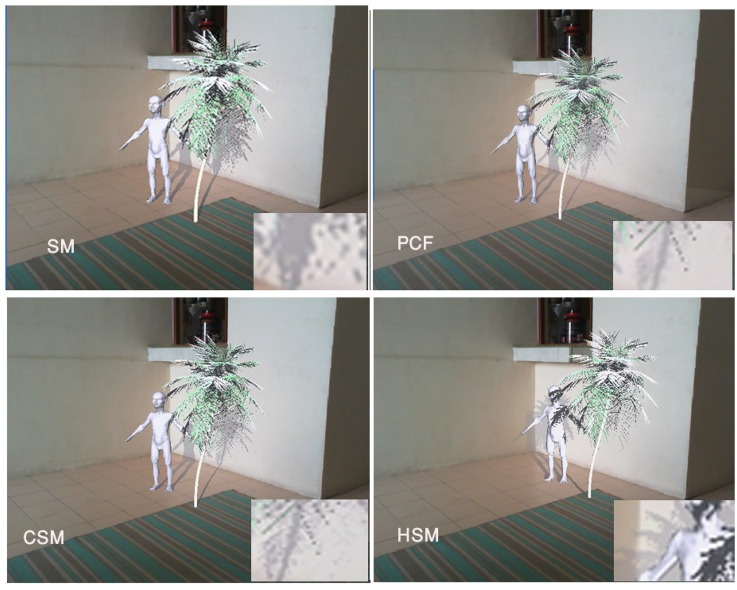
Different types of shadows on virtual and real objects simultaneously, top-left: Shadow Maps, top-right: PCF, down-left: CSMs, down-right: HSMs.

Fig 7 illustrates a scene including two virtual objects, a tree and a goblin. The virtual shadows of the tree are cast on the virtual goblin and the real wall, simultaneously. The shadow technique used in this picture is that of standard shadow maps with 512*512 resolution which does not produce adequate results. Applying PCF with 1024*1024 resolution on top-right side picture yields better results. Fig 7 (down-left) depicts the results of CSMs in the same scene. The virtual shadows are cast on the virtual and real environments, simultaneously. Compared to the standard shadow mapping, in the PCF, aliasing is removed and semi-soft shadows are obtained all of which make the environments more realistic. Fig 7 (down-right) shows the same scene using HSMs.

**Fig 8:**
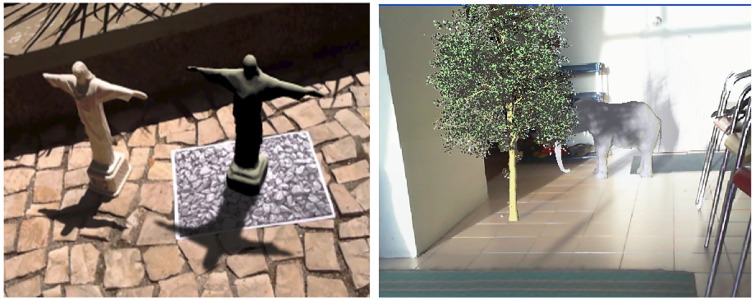
Left: Castro result, right: semi-soft shadows using HSMs on real and virtual objects.

Castro et al. [19] proposed a method to produce semi-soft shadows for AR systems. The method does not support self-shadowing as it can be observed on the base of the virtual statue (Fig 8 (left)). The result shows the technique projects shadows due to flat shadows which do not show any embossing on the stones. Fig 8 (right) is result of HSMs which cast virtual soft shadows on virtual and real environments perfectly.

### 0.9 Real-Time Reconstruction

The most difficult task facing this study is the reconstruction of real environments in real-time. LivePhantom helps reconstruct the phantoms in real-time. The techniques mentioned in the previous works did not tackle the issue of adding or removing objects form the scene in real-time. LivePhantom is able to create the new phantom by changing the scene but the new phantom is reconstructed within seconds. It depends on the complexity of the scene ranging from 1 ms to 1.45 seconds. It is more helpful to have shadows on added objects than to remove the shadows on omitted ones. The following figures reveal this ability as well.

**Fig 9:**
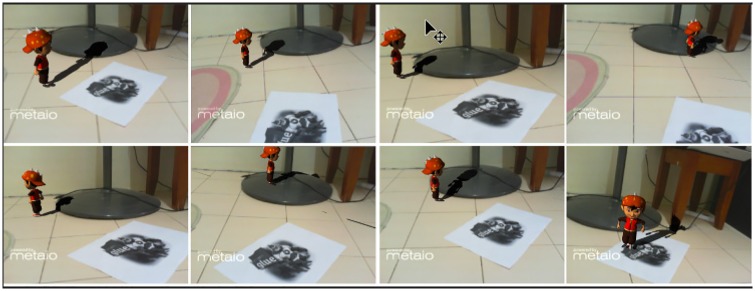
Virtual object walks on the stand of a fan.

Fig 9 is a scene that includes some real objects and a virtual character that can walk, run and jump. The character stops when facing obstacles; such as walls or any other real objects. The phantom which is created using LivePhantom is set as a mesh collider. As a result, all the real objects in the scene act as a collider, thus, the virtual character cannot pass through the real objects.

**Fig 10:**
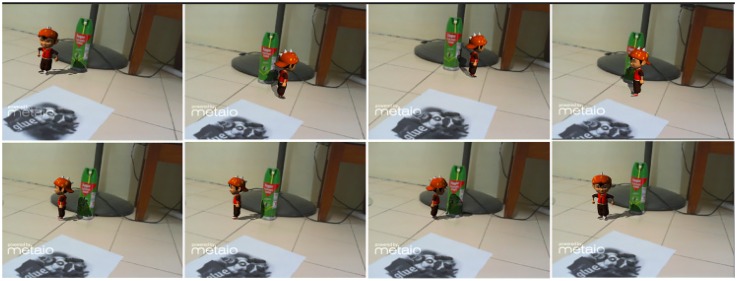
A simple real object is added in the environment.

**Fig 11:**
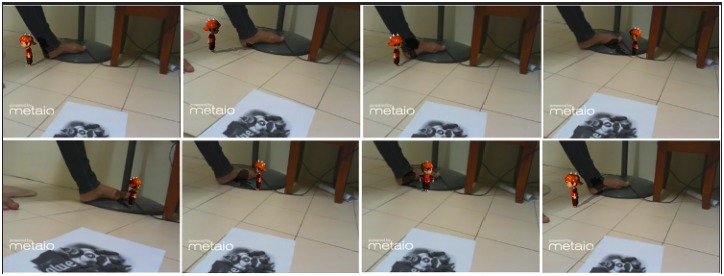
A complex real object is added in the environment.

**Fig 12:**
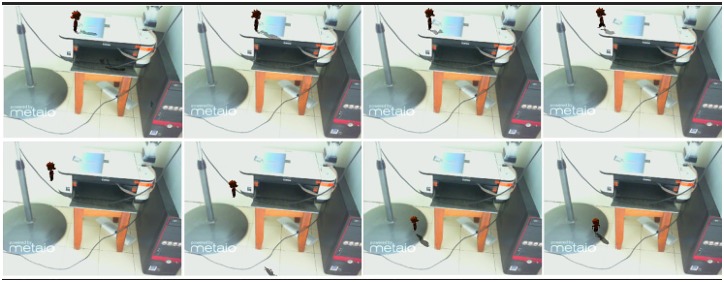
Step by step moving the augmented object in the real scene with an accurate interaction with the real environment (from left to right).

In Figs 10, 11 and 12 other real objects are added in real-time. Virtual shadows are cast on the added objects as well as the previous ones. An accurate shadow on real environments makes the LivePhantom technique more robust.

Reconstruction within seconds is one of the issues with LivePhantom but using a separate camera as the AR camera from the Kinect camera addresses this issue. After reconstructing the phantom, AR camera can move around the scene and change the view of AR system without recreating a new phantom. This is helpful for enhancing the rendering time.

Kinect cannot recognise far away objects as well as the closer ones due to using an infra-red ray. This forces us to use LivePhantom for indoor rendering.

### 0.10 Physical Interaction in Augmented Reality

At this juncture, it is necessary to demonstrate the robustness of the system for interactions occurring between real and virtual environments. All animated and non-animated objects can be utilized in the system. To further enhance the realism of the mixed system, any conflicts between the virtual and real objects needs to be prevented.

Different types of physical effects, e.g. rigid body, can be implemented on the phantoms in the form of a mesh collider. The invisible phantom in mixed reality environments further enhances the interactive capability of the environments. For instance, the virtual character can interact with the real environments. Fig 12 illustrates some parts of an animation in an AR system interacting with the real environment. The augmented object accurately interacts with the real environment. Shadows on the book over the printer can be seen very accurately. While walking, if the character passes from the surface of the printer it falls down. The shadows are precisely cast on the real environments during walking and falling down.

Table 1 shows that the difference between FPS during adding or removing objects can be ignored. In this Table “non” means when Kinect is not used, “single” means when the Kinect is fixed and “multi” means when the Kinect is moved around the scene. In general, LivePhantom increases rendering time by roughly 57.54%. The major difference shows itself while the Kinect camera’s position has changed. In some situations it increases to 71.55% or 24 FPS, depending on the complexity of the environments. Case 4 and 5 are the same scenario but in the case of static and dynamic virtual objects respectively. In Case 4, the virtual objects do not move. The result of FPS only back to the capturing a complex environment while reduce the FPS in Case 5 shows the affect of animation and controlling the animated objects using input device.

**Table 1:** Frame Per Second for different scenarios and different types of capturing.

Different Scenes	FPS (Viewpoints capturing)		
	Non	Single	Multi
Case 1 (Fig 9)	84.36	36.28	16.34
Case 2 (Fig 10)	84.36	35.47	16.11
Case 3 (Fig 11)	84.36	36.11	15.12
Case 4 (Fig 12)	84.36	35.84	14.02
without animation			
Case 5 (Fig 12)	84.36	35.51	12.35
with animation			

**Fig 13:**
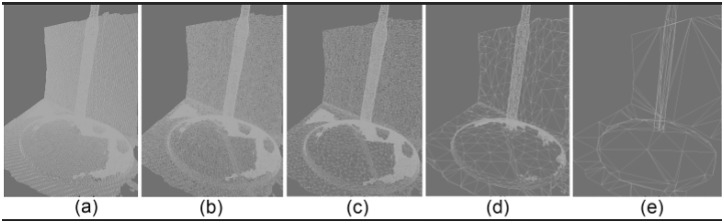
Reducing mesh; (a) 499994Tri#, (b): 71659 Tri#, (c): 17913Tri#, (d): 6231 Tri#, (e): 202 Tri#.

Reducing the quality of the phantoms could increase the FPS depending on the density of the meshes which are going to be used in the AR systems (Fig 13). Table 2 shows a comparison between different qualities of meshes in the AR system.

**Table 2:** Frame Per Second for different scenarios and different types of capturing.

Number of faces	FPS
8335263	4.23
735567	11.20
40724	18.34
7135	26.23
1791	38.78
560	49.31
208	61.89
97	70.91
43	75.94

The results of Table 2 are obtained using an Alienware laptop, Intel i7-2670QM CPU 2.20GHz and 8.0 GB RAM with Graphic Hardware GeForce 7025 NVIDIA nForce 630a.

Table 1 also shows that the complexity of the scene in the case of fixed Kinect camera is not important when it comes to the FPS. This is due to capturing all objects from a single side. Complex scenes cause low frame per second during moving the Kinect camera.

## Conclusion and Future Works

Shadows are from among the most salient parameters by means of which AR systems are rendered realistic. Exerting virtual shadows on mixed environments in real-time has been a major objective for the present research whereby AR realism is reinforced. Phantoms are considered as virtual shadows on real objects. Pre-reconstruction of real environments is the well-explored technique to cast virtual shadows on real objects for about ten years. But it suffers from using real-time reconstruction. In this study, reconstructing the real environments using Kinect aimed at creating real-time phantoms which were placed onto the real ones. This method is more accurate and quite fast compared to the other works which tend to generate phantoms in advance [18].

LivePhantom is proposed to generate real-time phantoms which do not necessarily have to be produced in advance. A Kinect camera captures environments by connecting neighbouring pixels in the induced point cloud for the phantom to be utilized as AR object. The 3D mesh reconstructs within milliseconds (less than 28 ms) demonstrating the technique to be suitable for real-time rendering. The phantoms can receive the virtual shadows as a simple virtual object on virtual environments but can be observed in real environments due to the transparency of the phantom under no more tolerance in real-time.

The phantoms generated using LivePhantom could be used as a collider. It means interaction between animation objects and real objects could be observed in the course of animating the virtual object.

In the case of the shadow technique, semi-soft shadows are taken into account to enhance the realism of AR environments. Some recent and widely used shadow techniques have been employed in AR system. Conventional Shadow Maps, PCF, CSMs and HSMs are applied in a same scene to highlight the capability of LivePhantom.

The results show that LivePhantom can conveniently be employed in real-time rendering environments. Employing different types of shadows and physical interaction shows that other phenomena such as illumination, animation and different types of visualisation can employ this technique. Moreover, it can be used for various reconstructions in engineering applications.

As mentioned earlier, LivePhantom reconstructs 3D meshes within seconds. The main issue with LivePhantom is the lack of casting accurate shadows on real objects which move quickly. Enhancing the technique to generate the sufficiently high quality phantoms having fast rendering time and thus increasing the FPS is the issue to improve the current LivePhantom. Implementing the LivePhantom technique in outdoor AR system is another issue which must be taken into consideration.

The interaction between virtual and real objects, such as exerting the colour, influence of real objects on virtual ones and vice versa can largely enhance the realism. Focusing on the colour sensor of a simple Kinect camera and Radiosity and Ray-tracing techniques is the next step to enhance the interaction between virtual objects and real environments.

It is hoped that the present study could broaden researchers’ perspectives for applying this technique in both computer graphics and other related disciplines.
